# Quantitative EEG signatures in patients with and without epilepsy development after a first seizure

**DOI:** 10.1002/epi4.13128

**Published:** 2025-03-04

**Authors:** Marysol Segovia‐Oropeza, Erik Hans Ulrich Rauf, Ev‐Christin Heide, Niels K. Focke

**Affiliations:** ^1^ Clinic of Neurology University Medical Center Göttingen Göttingen Germany; ^2^ University of Göttingen Göttingen Germany

**Keywords:** biomarkers, resting state, routine electroencephalography, source‐reconstruction, unprovoked seizures

## Abstract

**Objective:**

Diagnosing epilepsy after a first unprovoked seizure in the absence of visible epileptogenic lesions and interictal epileptiform discharges (IED) in the electroencephalogram (EEG) is challenging. Quantitative EEG analysis and functional connectivity (FC) have shown promise in identifying patterns across epilepsy syndromes. Hence, we retrospectively investigated whether there were differences in FC (imaginary part of coherency) and spectral band power in non‐lesional, IED‐free, unmedicated patients after a first unprovoked seizure in contrast to controls. Further, we investigated if there were differences between the patients who developed epilepsy and those who remained with a single seizure for at least 6 months after the first seizure.

**Methods:**

We used 240 s of resting‐state EEG (19 channels) recordings of patients (*n* = 41) after a first unprovoked seizure and age and sex‐matched healthy controls (*n* = 46). Twenty‐one patients developed epilepsy (epilepsy group), while 20 had no further seizures during follow‐up (single‐seizure group). We computed source‐reconstructed power and FC in five frequency bands (1 ± 29 Hz). Group differences were assessed using permutation analysis of linear models.

**Results:**

Patients who developed epilepsy showed increased theta power and FC, increased delta power, and decreased delta FC compared to healthy controls. The single‐seizure group exhibited reduced beta‐1 FC relative to the control group. In comparison with the single‐seizure group, patients with epilepsy demonstrated elevated delta and theta power and decreased delta FC.

**Significance:**

Source‐reconstructed data from routine EEGs identified distinct network patterns between non‐lesional, IED‐free, unmedicated patients who developed epilepsy and those who remained with a single seizure. Increased delta and theta power, along with decreased delta FC, could be a potential epilepsy biomarker. Further, decreases in beta‐1 FC after a single seizure may point toward a protective mechanism for patients without further seizures.

**Plain Language Summary:**

After a first seizure, some people develop epilepsy, while others do not. We looked at brain activity in people who had a seizure but showed no clear signs of epilepsy. By comparing those who later developed epilepsy to those who did not, we found that certain slow brain wave patterns (delta and theta) might indicate a higher risk of developing epilepsy. This could help doctors identify high‐risk patients sooner.

**Key points**

- Unmedicated, non‐lesional patients who developed epilepsy after a first seizure have increased delta and theta power along with increased theta FC.
- Increased power in delta and theta bands together with decreased delta FC could be a possible biomarker for epilepsy.
- Unmedicated patients who remain with one unprovoked seizure have decreased beta 1 FC.

## 1 INTRODUCTION

An epileptic seizure is a transient occurrence of signs and/or symptoms due to excessive or synchronous neuronal activity in the brain.^1^ An unprovoked seizure happens in the absence of an immediate precipitating event, and the risk of recurrence is highest (21%–45%) within the first 2 years following the initial seizure.^2^, ^3^, ^4^ Having at least two unprovoked seizures occurring less than 24 h apart from each other is one of the criteria to meet the epilepsy diagnosis. If only a single unprovoked seizure has occurred, a probability greater than 60% of further seizures in the following 10 years warrants the diagnosis and usually, the initiation of anti‐seizure medication (ASM).^1^ Factors that increase the probability are structural lesions or epileptiform activity in the EEG.^5^, ^6^, ^7^ Studies in adults who have experienced a first unprovoked seizure show that the yield of the initial EEG detecting interictal epileptic discharges (IEDs) ranges from 12% to 55%.^8^, ^9^, ^10^ For patients with normal routine EEG (10–20 system) and non‐lesional MRI that are being assessed for epilepsy, a more expensive and time‐consuming sleep‐deprived EEG or a prolonged EEG/video‐EEG may be necessary. While automated spike detection could reduce costs, expert confidence is still low.^11^, ^12^ Additionally, there is an 8% incidence of spikes among non‐epileptic patients.^13^, ^14^ Another challenge is ruling out differential diagnoses, as epilepsy misdiagnoses in adults occur in 5%–26% of cases,^15^, ^16^, ^17^ often resulting in inappropriate pharmacological treatment.

Epilepsy is a brain network disease characterized by an enduring predisposition to generate seizures^18^ which originate and spread in networks.^19^, ^20^ Functional connectivity (FC), the temporal coincidence of spatially distinct neurophysiological events,^21^, ^22^ is one method to study neuronal interactions, although other approaches have also been successful in describing epileptic brain dynamics.^23^

Research using magnetoencephalography (MEG) and EEG has shown pathological networks during the epileptic brain's resting state. Most studies report widespread increased FC and power in delta to beta 1 frequency bands^24^, ^25^, ^26^, ^27^ in patients with idiopathic generalized epilepsy (IGE) compared to healthy controls. In patients with focal epilepsy, findings vary but commonly include increased FC in the epileptogenic zone^28^, ^29^, ^30^, ^31^, ^32^ effectively helping to localize seizure onset zones.^33^

Quantitative EEG analysis after an initial seizure has been limited. One sensor‐space study examined network differences between patients who had a first seizure and patients who later developed epilepsy.^34^ The epilepsy patients showed increased FC and power in the theta band. In a source‐space study, the changes in connectivity and power were specific to the alpha and beta bands.^35^ Another group developed a prediction model using EEG‐derived functional network characteristics, in order to distinguish children with partial epilepsy from those who experienced a single seizure.^36^ Important limitations addressed by these studies are that they included heterogeneous participants with radiological abnormalities, relevant comorbidities, differential diagnoses, and CNS active medication, which strongly affect connectivity and power.^25^, ^34^ To overcome these obstacles, more source‐space studies are needed. Canonical head models could support source‐projection in clinical settings, enhancing anatomical precision and improving the signal‐to‐noise ratio,^37^, ^38^ thereby advancing our understanding of brain changes after a first seizure.

Identifying biomarkers to detect high‐risk epilepsy patients after a first unprovoked seizure could enable immediate treatment, reducing epilepsy‐related injuries and morbidity, and decreasing unnecessary ASM treatment, thereby enhancing patient quality of life. Therefore, this work investigated the changes in resting‐state FC and power in a non‐medicated non‐lesional cohort that had a first seizure. The central hypothesis of this study posits that patients predisposed to developing epilepsy following an initial seizure, will exhibit different functional connectivity (FC) and EEG power signatures in comparison with those who will remain with a single seizure. Further, we hypothesize that EEG signatures in patients who do not develop epilepsy are more similar to healthy subjects.

## 2 MATERIALS AND METHODS

### 2.1 Participants

We retrospectively reviewed the clinical database of patients presenting with a first unprovoked seizure at Göttingen University Medical Center, Germany, from January 2010 to June 2023. Exclusion criteria included a known present or past neurological and psychiatric disorder, major medical comorbidity, substance abuse, pregnancy, brain lesions, history of epilepsy, a previous epilepsy differential diagnosis (e.g., syncope, transient ischemic attack, and psychogenic seizure), and the administration of ASM before the electroencephalographic evaluation. Inclusion required an available EEG recorded within 6 months post‐seizure showing no interictal epileptic discharges (IEDs). We utilized all accessible clinical data for follow‐up purposes, setting a minimum duration of 6 months or until a second seizure occurred. Patients with only one seizure were classified into the single‐seizure group, while those with a second seizure were categorized under the epilepsy group as per International League Against Epilepsy (ILAE) guidelines. When the type of epilepsy was not specified in the clinical report, an experienced neurologist (R.E.H.U. or H.E.C) reviewed the clinical data, including EEG, MRI, and reports, to determine the epilepsy syndrome. The study and the recruitment of healthy subjects were approved by the local ethics committee (reference numbers: 22.04.21 and 02.05.21), and informed consent was obtained from the healthy subjects. For more clinical details, see Table 1.

**TABLE 1:** Summary of patients and healthy controls characteristics.

	Controls (*n* = 46)	Single‐seizure patients (*n* = 20)	Epilepsy patients (*n* = 21)
Age (years)			
Mean ± STD	36.08 ± 15.01	43.95 ± 15.75	40.19 ± 16.87
Range	18–72	21–70	18–68
Gender	25 males, 21 females	10 males, 10 females	15 males, 6 females
Days from seizure to EEG			
Mean ± STD		45.5 ± 60.11	13.95 ± 22.99
Range		1–180	1–74
Type of first seizure			
Generalized (%)		18.1	19.1
Focal (%)		22.7	9.5
Unclassified (%)		59.2	71.4
Type of epilepsy			
Generalized (%)			23.8
Focal (%)			57.14
Unclassified (%)			19.04

### 2.2 EEG signal recording and processing

The 20‐min, resting state EEG was done using the standard 10–20 system (19 electrodes) using a digital electroencephalograph (Natus®, Nicolet/Xltek). Subjects were instructed to close their eyes but not to sleep. The data were exported from the Natus® system with a sampling frequency of 500 Hz. The data were processed as described elsewhere^39^ using Fieldtrip^40^ and Matlab (v9.0, R2017b, MathWorks). In brief, we applied a first‐order Butterworth band pass filter (1–70 Hz) and a band‐stop filter to remove noise at 50, 100, and 150 Hz. The data were down‐sampled (150 Hz) and segmented into trials of 10 s length. Each trial was visually inspected and rejected if noise (e.g., muscle artifacts, sensor jumps) were present. Cardiac artifacts and eye movements were removed by independent component analysis. Later, each trial was scored for vigilance according to the American Academy of Sleep Medicine (https://aasm.org/). Twenty‐four trials (240 s)^39^ of processed EEG signal scored as awake were randomly selected per subject for further analysis.

### 2.3 Source reconstruction

To conduct source‐level analysis, we used a canonical head model created from an MRI template obtained from 225 normal‐control adults originally scanned with 3T T1+FLAIR. The images were nonlinearly transformed to an MNI‐space similar template using ANTs/SyN transformations.^41^ This template image was passed through Freesurfer^42^ and SUMA^43^ to yield a canonical head model. This procedure produced 1169 common vertices per hemisphere as EEG source points, with vertex‐based correspondence across subjects. For each vertex of the cortical mesh, a lead field matrix was calculated. Source reconstruction was done using dynamic imaging of coherent sources as a beamformer method^44^ and it was performed separately for every frequency band.

### 2.4 Spectral power and connectivity

An absolute spectral power and cross‐spectral density analysis was carried out on the EEG sensor level using Fieldtrip.^40^ The relative power values were obtained for five frequency bands: delta (2 ± 2 Hz), theta (6 ± 2 Hz), alpha (10 ± 2 Hz), beta 1 (16 ± 4 Hz), and beta 2 (25 ± 4 Hz). There are many metrics to study functional connectivity which have been described and reviewed in the literature.^45^, ^46^, ^47^ We used the absolute imaginary part of coherency (ImCoh) as our connectivity measure, because it reduces the influence of the zero‐lag component of interactions between signals, thus minimizing the influence of potentially spurious correlations,^46^, ^48^ which makes this metric less affected by potential field spread. Further, ImCoh has already proven useful for identifying epileptogenic zones^49^, ^50^ and detecting network changes in patients with non‐lesional focal^31^ and idiopathic generalized epilepsy.^51^

After beamforming, ImCoh values were calculated for every frequency band between all pairs of vertices (2338 vertices). As a result, an individual, symmetrical, and weighted matrix was constructed for each frequency band. For FC, the strength of each vertex was estimated by averaging the strength of all its connections to obtain a single ImCoh value per vertex (vertex‐based connectivity). These values were later used for statistical analyses. The localization of the vertices was labeled with the use of the Desikan‐Killiany atlas.^52^ We also calculated global ImCoh values by averaging the vertex‐based values for each frequency band and subject, yielding a single “global” measure for each participant and frequency band.

### 2.5 Statistics

The time between the seizure and EEG recording, follow‐up duration, and age were non‐normally distributed (Lilliefors, *p* < 0.05); therefore, we used the Mann–Whitney *U*‐test for comparisons. A Chi‐square test was used to assess frequencies of sex within groups.

Group differences in power and ImCoh were analyzed using Permutation Analysis of Linear Models (PALM), a nonparametric statistical tool (fsl.fmrib.ox.ac.uk/fsl/fslwiki/PALM). To contrast the global and vertex‐based data, we used *t*‐tests between every pair of groups. A general linear model (GLM) was computed for every permutation with power and ImCoh metrics as dependent variables. Sex and demeaned age were included in the GLMs as regressors. This process was repeated 5000 times with shuffled subjects and tail approximation, resulting in empirical distributions from which *p*‐values were generated. In the vertex‐based analysis, a correction for multiple comparisons on the cluster level using threshold‐free cluster enhancement (TFCE) was implemented.^53^ Family‐wise error correction (FWE) was performed for *p*‐values within each group contrast. Effect sizes (Cohen's *d*) were calculated for vertex‐based and global group comparisons. An effect size of *d* = 0.2 is considered to be small, *d* = 0.5 medium, and *d* = 0.8 large.^54^ *p*‐values were log‐transformed and are indicated as −log10 (*p*‐value). We used a significance threshold of –log *p* = 1.3 (~*p* ≤ 0.05). *p*‐values per vertex and metric were labeled according to the Desikan‐Killiany surface‐based map,^52^ which allowed an analysis according to anatomical regions.

## 3 RESULTS

The epilepsy group (*n* = 21, mean age ± SD = 40.19 ± 16; 15 females) and the single‐seizure group (*n* = 20, mean age ± SD = 43.95 ± 15; 10 females) were matched for age and sex with healthy controls (*n* = 46, mean age ± SD = 36 ± 15; 21 females). There were neither significant differences in sex ($X_{\left(1\right)}^{2}$ = 1.74; *p* = 0.18) nor in age (*U* = −0.75, *p* = 0.44) between patients with epilepsy and the control group. Sex was equally distributed ($X_{\left(1\right)}^{2}$ = 0.10; *p* = 0.74) in the single‐seizure group and the controls, but we found a trend‐level difference in age (*U* = −1.75, *p* = 0.07). Therefore, we included age and sex as regressors of no interest in all our analyses. The average time between the seizure and the EEG was 13.95 days (SD = 22.99) and 45.5 days (SD = 60.11) for the epilepsy and single‐seizure groups, respectively. We did not find these differences to be significant (*U* = 1.48, *p* = 0.13). The follow‐up duration between the epilepsy group (mean days ± SD = 657.33 ± 828) and the single‐seizure group (mean days ± SD = 436.9 ± 466) was not significantly different (*U* = −0.86, *p* = 0.38). Similarly, there were no significant differences in sex as well as sex ($X_{\left(1\right)}^{2}$ = 1.97; *p* = 0.15) and age (*U* = 0.86, *p* = 0.38).

In this study, according to the inclusion criteria, the initial routine EEG used for analyzing FC and power, did not contain any IEDs in any of the subjects. Follow‐up EEGs, conducted as part of the medical assessment, were available for 38 subjects (93% of the cohort). In these EEGs, used solely for classification purposes, none of the patients in the single‐seizure group showed IEDs. Conversely, 76.1% (16 patients) of the epilepsy group exhibited IEDs. The subjects classified as having epilepsy included 5 patients with idiopathic generalized epilepsy (IGE) and 12 with focal epilepsy. Among those with focal epilepsy and IEDs during the follow‐up period, six had IEDs located in the frontotemporal region, three in the temporal region, one in the frontocentral region, and two in other regions. Additionally, 83.3% (*n* = 10) of those with focal epilepsy showed likely left‐sided epileptogenesis. Four patients had unclassified epilepsy types. For more details, see Table 1.

### 3.1 Epilepsy group versus healthy controls

The EEG power in patients with epilepsy shows widespread increases in the delta and theta bands. Regarding FC, we found regional increased theta and decreased delta in contrast to healthy controls.

**FIGURE 1:**
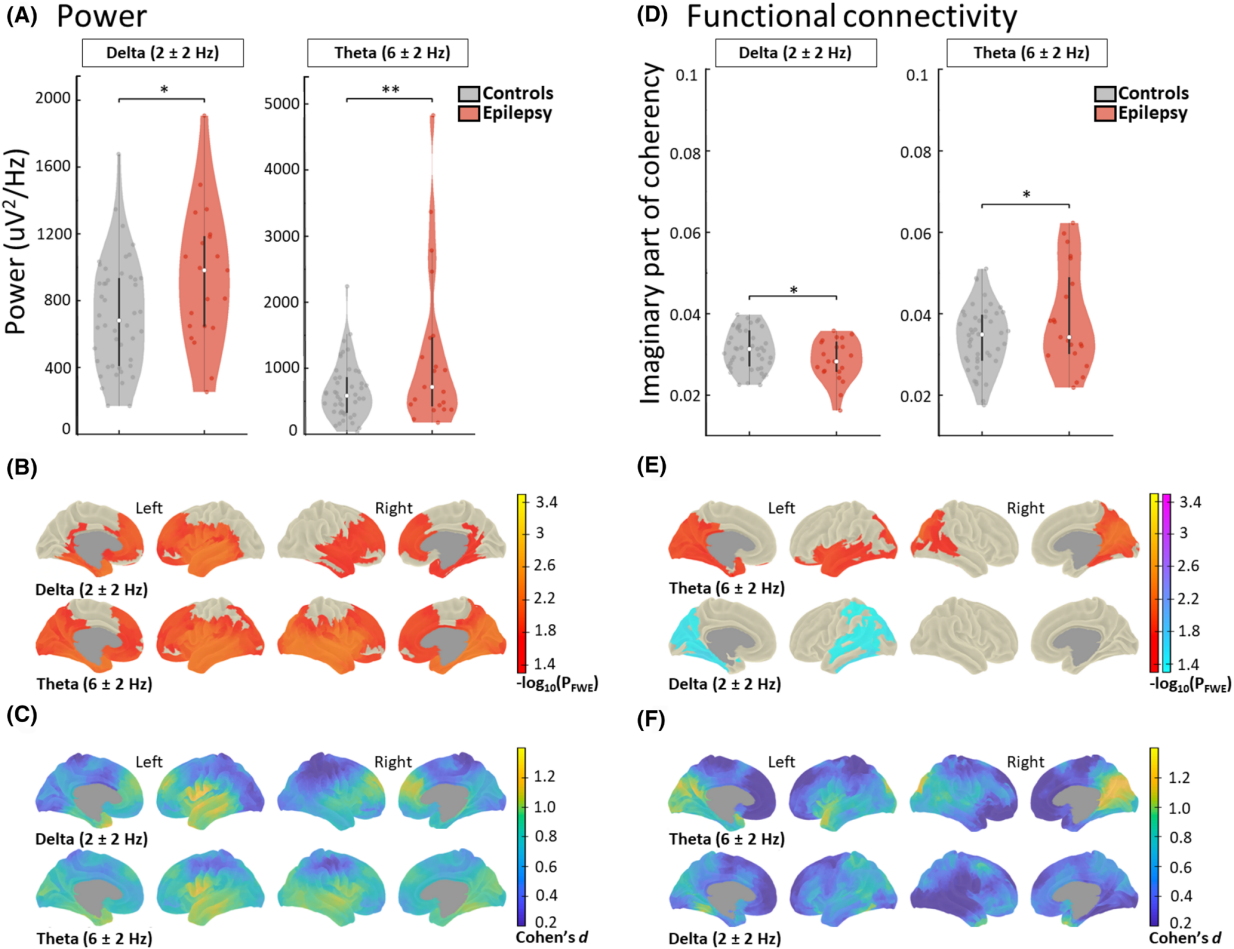
Electroencephalography analysis of power and functional connectivity of epilepsy patients against healthy controls. (A) Individual global power and (D) connectivity (imaginary part of coherency) of healthy controls (*n* = 46) and patients (*n* = 21) that developed epilepsy. The density, medians, and interquartile range of the data from frequency bands that had statistically significant group differences are shown (*p < 0.05; **p < 0.01). (B, E) Vertex level comparisons between the groups where increases are shown in red to yellow and decreases in blue to purple. The significance is coded at a −log_10*p*_ threshold of 1.3 (equivalent to *p* < 0.05 familywise error [FWE] corrected). (C, F) The blue to yellow plots show the standardized effect sizes which were derived from the *t*‐values (Cohen's *d*; *d* = 0.2, small effect; *d* = 0.5, medium effect and *d* ≥ 0.8, large effect).^54^ Statistical comparisons that did not yield significant results are not shown. We used permutation‐based analysis of linear models for global and vertex analysis with age and sex as covariates of no interest. Probability values were familywise error (FWE) corrected for whole‐brain analysis within each frequency band.

Specifically, the patients who developed epilepsy had higher global power in the delta (*p* = 0.01, *d* = 0.62) and theta (*p* = 0.007, *d* = 0.71) frequency bands (Figure 1A). The vertex level analysis localized bilateral increased power not only in frontotemporal regions (See Table 2, Figure 1B,C) but also in areas like the inferior parietal gyrus (*p* = 0.015, *d* = 0.84), circular insular sulcus (*p* = 0.032, *d* = 0.76), and anterior cingulate (*p* = 0.026, *d* = 0.91; Figure 1B,C). For a list with all the significant areas per frequency band, see Table S1. The epilepsy group also had a widespread, bilateral increase in power in the theta band compared to controls (Figure 1B,C). The regions with the highest number of significant vertices (Table 2) were the superior temporal sulcus (*p* = 0.009, *d* = 0.85), the superior frontal gyrus (*p* = 0.025, *d* = 0.72), and the anterior cingulate gyrus and sulcus (*p* = 0.025, *d* = 0.66). Interestingly, for ImCoh, theta and delta bands showed a divergent pattern. While delta band ImCoh was lower in epilepsy patients at the global (*p* = 0.03, *d* = 0.53) and vertex level (Figure 1D–F), theta FC was globally (*p* = 0.03, *d* = 0.52) and regionally increased in comparison with controls (Figure 1D–F; Table 1).

**TABLE 2:** Regional group differences in vertex‐based power and functional connectivity analysis.

Group comparison	Freq. band	Regions (Desikan‐Killiany atlas)	*pFWE* (Min)	log_10_ (pMax)	Sig. vertices	Cohen's *d* (Max)
*Power*						
Epilepsy > Controls	Delta	Left Superior Temporal Sulcus	0.011	1.95	29	0.90
		Left Superior Frontal Gyrus	0.018	1.74	21	0.86
		Right Anterior Cingulate Gyrus and Sulcus	0.026	1.58	20	0.91
		Left Superior Circular Insular Sulcus	0.013	1.88	17	0.82
		Right Superior Frontal Gyrus	0.026	1.58	16	0.92
		*Plus 92 regions*				
	Theta	Right Superior Temporal Sulcus	0.010	2.01	45	0.86
		Left Superior Temporal Sulcus	0.011	1.94	44	0.87
		Left Inferior Parietal Gyrus, Supramarginal Part	0.014	1.87	21	0.73
		Right Anterior Cingulate Gyrus and Sulcus	0.026	1.59	21	0.66
		Left Superior Circular Insular Sulcus	0.012	1.92	20	0.87
		*Plus 133 regions*				
Epilepsy > Single seizure	Delta	Left Superior Frontal Gyrus	0.011	1.93	54	1.15
		Left Superior Temporal Sulcus	0.009	2.03	43	1.16
		Right Superior Frontal Gyrus	0.024	1.61	43	1.04
		Left Postcentral Sulcus	0.013	1.88	32	0.89
		Left Central Sulcus	0.010	1.92	31	0.95
		*Plus 118 regions*				
	Theta	Left Superior Frontal Gyrus	0.039	1.40	31	0.95
		Right Superior Temporal Sulcus	0.034	1.46	29	0.81
		Right Superior Frontal Gyrus	0.033	1.47	26	0.92
		Right Superior Parietal Gyrus	0.034	1.46	23	0.72
		Right Intraparietal Sulcus and Transverse Parietal Sulcus	0.034	1.46	21	0.75
		*Plus 82 regions*				
*Imaginary part of coherency*						
Epilepsy > Controls	Theta	Left Calcarine Sulcus	0.024	1.62	19	0.89
		Left Superior Temporal Sulcus	0.029	1.54	18	0.75
		Right Calcarine Sulcus	0.014	1.87	16	1.03
		Right Medial Occipital Temporal Gyrus, Lingual Part	0.015	1.82	16	0.95
		Left Parieto‐Occipital Sulcus	0.024	1.63	16	0.87
		*Plus 60 regions*				
Epilepsy < Controls	Delta	Left Superior Temporal Sulcus	0.041	1.39	23	0.70
		Left Medial Occipital Temporal Sulcus and Lingual Sulcus	0.026	1.58	11	0.92
		Left Calcarine Sulcus	0.036	1.44	11	0.71
		Left Lateral Occipital Temporal Gyrus, Fusiform Part	0.026	1.58	9	0.95
		Left Parieto‐Occipital Sulcus	0.035	1.46	9	0.68
		*Plus 28 regions*				
Single seizure < Controls	Beta 1	Right Superior Frontal Gyrus	0.010	1.96	29	0.93
		Right Superior Temporal Sulcus	0.009	2.01	23	0.87
		Right Superior Frontal Sulcus	0.010	1.97	21	1.01
		Right Middle Frontal Gyrus	0.010	1.97	19	0.96
		Right Anterior Cingulate Gyrus and Sulcus	0.011	1.95	18	0.98
		*Plus 60 regions*				
Single seizure > Epilepsy	Delta	Left Superior Temporal Sulcus	0.026	1.58	26	1.21
		Left Lateral Occipital Temporal Gyrus, Fusiform Part	0.023	1.62	12	1.39
		Left Medial Occipitotemporal Sulcus and Lingual Sulcus	0.024	1.60	11	1.23
		Left Middle Occipital Gyrus	0.027	1.55	8	1.04
		Left Inferior Occipital Gyrus and Sulcus	0.027	1.56	7	1.11
		*Plus 11 regions*				

*Note*: This table shows the five regions with the highest number of significant vertices based on power and connectivity group comparisons (p‐FWE values). The number of other significantly different regions is reported for every contrast and frequency band, within the 188 regions of the Desikan‐Killiany atlas.^52^ The corresponding maximal −log_10*p*_ value, number of vertices in that region, and maximal Cohen's *d* ^54^ are also reported. A complete table of regions and their statistically significant results is provided in Table S1.

The specific areas of theta FC increase (Figure 1E,F), were not only posterior regions like the cuneus gyrus (*p* = 013, *d* = 1.01), but also the precuneus (*p* = 0.013, *d* = 1.07) and superior temporal sulcus (*p* = 0.028, *d* = 0.74) among others. The delta decrease was specific to the left hemisphere. Some of the areas with the highest number of significant vertices were the superior temporal sulcus (*p* = 0.041, *d* = 0.70), lingual sulcus (*p* = 0.026, *d* = 0.91), and the calcarine sulcus (0.036, *d* = 0.71) (Figure 1E,F). No significant differences in power or FC were found across other frequency bands.

As part of a subanalysis, we compared the ipsilateral and contralateral hemispheres (with reference toward the laterality of the presumed epileptogenic focus) of patients with focal epilepsy (*n* = 12). Later, we compared those with left‐sided epileptogenesis (*n* = 10) to controls (data not shown). No significant differences were found in power or ImCoh across any of these comparisons (Figure S1A; Table S2). Additionally, we assessed differences between patients who developed IGE (*n* = 5) and those with focal epilepsy (*n* = 12). In the global analysis, we observed lower beta 1 FC in the IGE group (*p* = 0.01, *d* = 1.6), specifically in the right hemisphere (Figure S1B; Table S1). The IGE group also showed higher FC in the theta band in the right temporal region (Figure S1B; Table S1).

### 3.2 Single‐seizure group versus healthy controls

**FIGURE 2:**
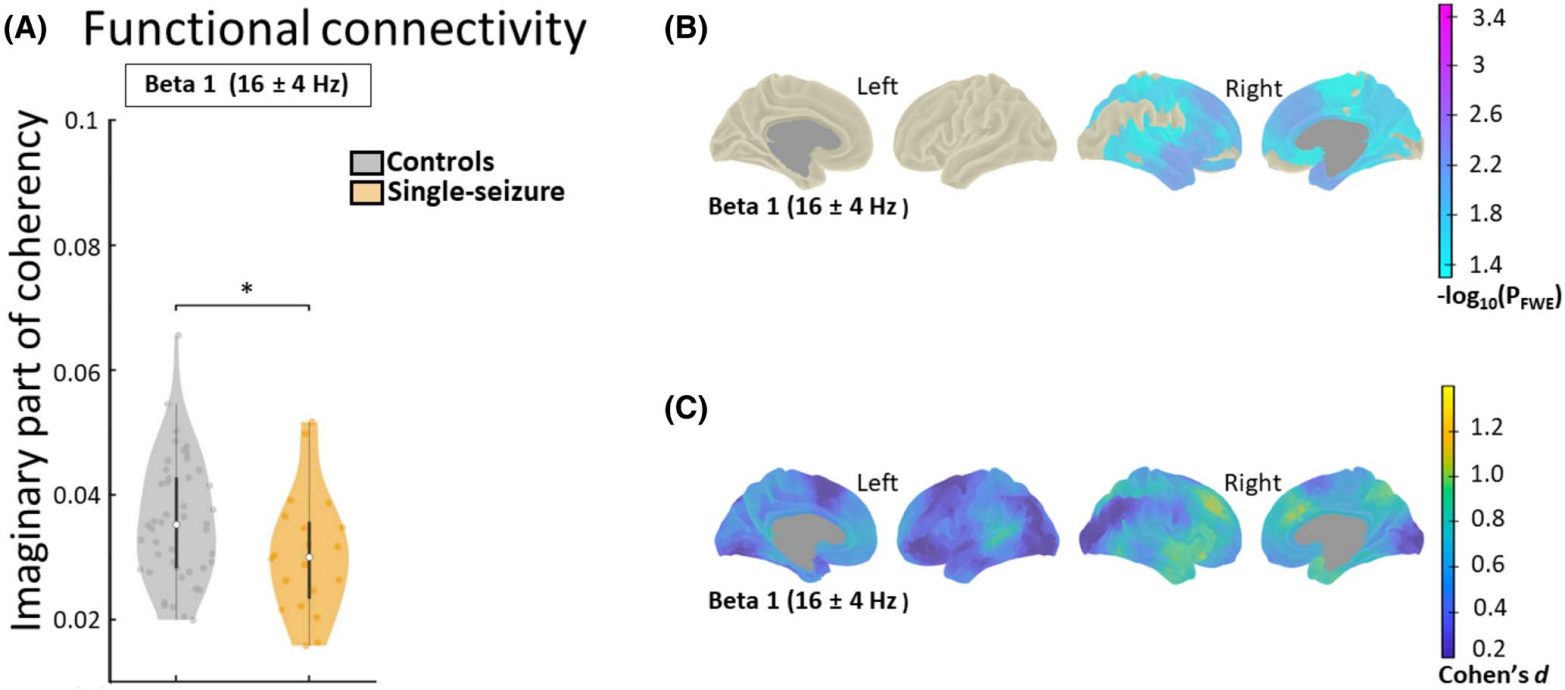
Electroencephalography analysis of functional connectivity of single‐seizure patients against healthy controls. (A) Individual global connectivity (imaginary part of coherency) comparison of controls (*n* = 46) and patients that remained with one seizure (*n* = 20). Shown are the density of the data, group medians, and interquartile range per frequency band. Statistically significant group differences are shown (*p < 0.05). (B) Vertex level comparisons between the groups where the decrease is shown in blue to purple. The significance is coded at a −log_10*p*_ threshold of 1.3 (equivalent to *p* < 0.05 familywise error [FWE] corrected). (C) The blue to yellow plot shows the standardized effect size which was derived from the *t*‐values (Cohen's *d*; *d* = 0.2, small effect; *d* = 0.5, medium effect and *d* ≥ 0.8, large effect).^54^ Statistical comparisons that did not yield significant results are not shown. We used permutation‐based analysis of linear models for global and vertex analysis with age and sex as covariates of no interest. Probability values were familywise error (FWE) corrected for whole‐brain analysis within each frequency band.

Patients with only one seizure who did not develop epilepsy showed decreased FC exclusively in the beta 1 band, with no other significant differences in power or FC noted. The decrease in beta 1 had a medium effect size (*p* = 0.020, *d* = 0.56, Figure 2A). In the vertex level, the decrease was localized in the right hemisphere (Figure 2B,C) with intermediate to large effect sizes. A bilateral pattern was only present in the uncorrected data (*p*_uncorr_ < 0.05). Significant findings included areas in the frontal lobe such as the superior gyrus (*p* = 0.01, *d* = 0.93) and sulcus (*p* = 0.01, *d* = 1.01), as well as the temporal lobe's superior temporal sulcus (*p* = 0.009, *d* = 0.87) as detailed in Table 2.

### 3.3 Epilepsy group versus single‐seizure group

The epilepsy patient's group was characterized by higher power in delta and theta bands. Meanwhile, the single‐seizure group showed higher delta FC.

**FIGURE 3:**
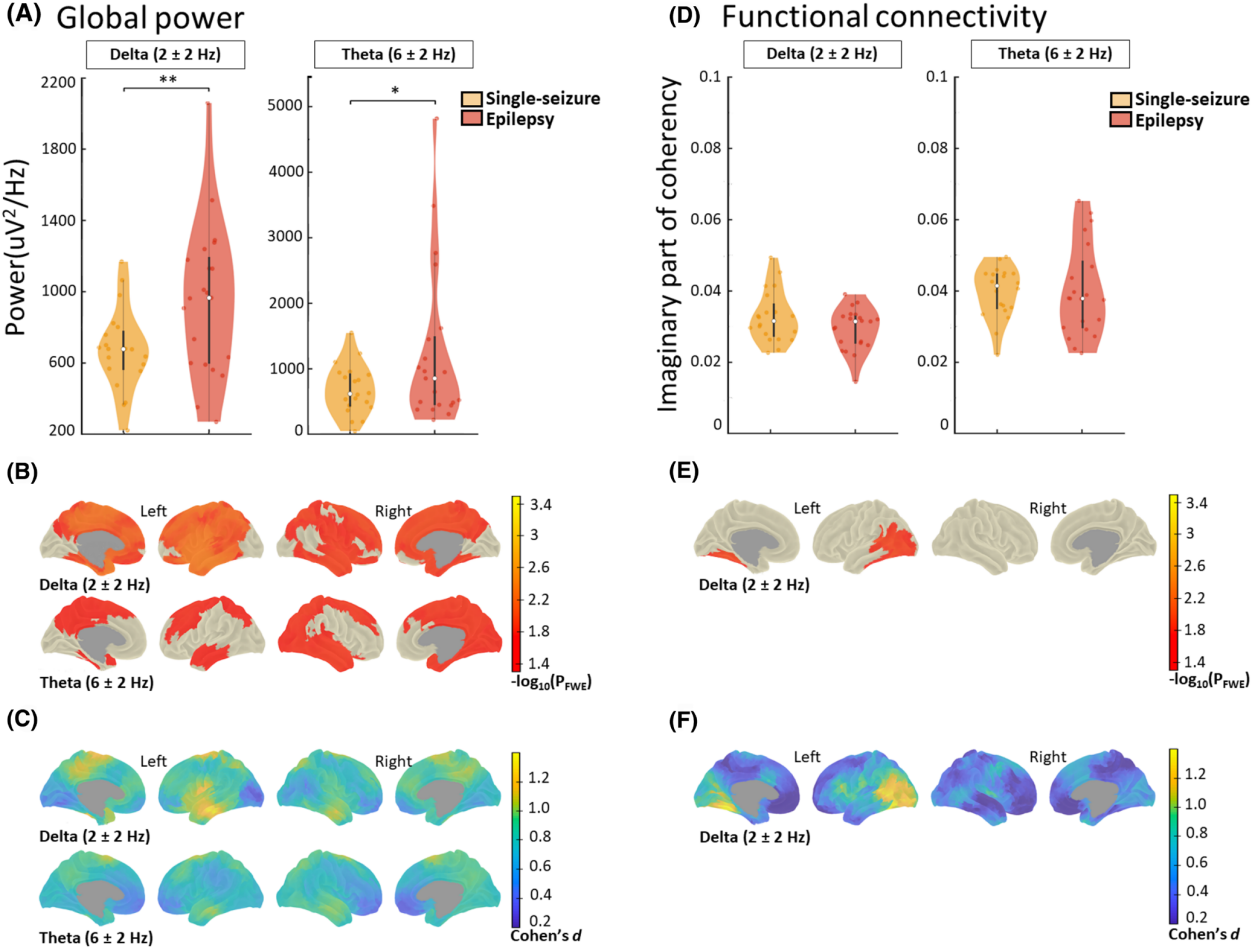
Electroencephalography analysis of power and functional connectivity of patients that developed epilepsy against patients that remain with a single seizure. (A) Individual global power and (D) connectivity (imaginary part of coherency) of epilepsy patients (*n* = 21) and patients with a single seizure (*n* = 20). The density, medians, and inter‐quartile range of the data from frequency bands that had statistically significant group differences are shown (*p < 0.05; **p < 0.01). (B, E) Vertex level comparisons between the groups where increases are shown in red to yellow. The significance is coded at a −log_10*p*_ threshold of 1.3 (equivalent to *p* < 0.05 familywise error [FWE] corrected). (C, F) The blue to yellow plots show the standardized effect sizes which were derived from the *t*‐values (Cohen's *d*; *d* = 0.2, small effect; *d* = 0.5, medium effect and *d* ≥ 0.8, large effect).^54^ Statistical comparisons that did not yield significant results are not shown. We used permutation‐based analysis of linear models for global and vertex analysis with age and sex as covariates of no interest. Probability values were familywise error (FWE) corrected for whole‐brain analysis within each frequency band.

Namely, epilepsy patients exhibited higher global power in both delta (*p* = 0.008, *d* = 0.79) and theta (*p* = 0.030, *d* = 0.60) bands, with widespread bilateral increases at the vertex level (Figure 3A–C; Table 2). Notable increases included the left superior gyrus (*p* = 0.011, *d* = 1.15) and the right superior gyrus (*p* = 0.024, *d* = 1.04) in the frontal lobe, and generally higher delta power in the parietal lobe (Table S1). Theta power was elevated across all lobes, with significant vertices in the left superior frontal gyrus (*p* = 0.039, *d* = 0.95), right superior temporal sulcus (*p* = 0.034, *d* = 0.81), and right superior frontal gyrus (*p* = 0.033, *d* = 0.92).

In the FC analysis, there were no differences at the global level (Figure 3D). At the vertex level, the epilepsy patients had lower delta FC in the left hemisphere (Figure 3E,F). Among the areas that showed the higher number of significant vertices were the occipitotemporal gyrus (*p* = 0.023, *d* = 1.39) and sulcus (*p* = 0.024, *d* = 1.23), and the superior temporal sulcus (*p* = 0.026, *d* = 1.21). For a list of all the significant areas per frequency band, see Table S1.

## 4 DISCUSSION

We conducted a study on the EEG network profiles of drug‐naïve, non‐lesional patients following their first seizure. The goal was to identify whether those who later developed epilepsy displayed distinct FC and power patterns before experiencing a second seizure compared to those without subsequent seizures. Further, we aimed to determine if single‐seizure patients exhibited abnormal networks compared to healthy subjects.

The single‐seizure patients exhibited lower beta 1 FC compared to controls. Additionally, the epilepsy group showed higher delta and theta power, and lower delta FC than the single‐seizure patients.

Few studies have investigated the network changes in the context of a first seizure, particularly those comparing patients with healthy controls. To the best of our knowledge, the decreased beta 1 FC observed in patients who remain with a single seizure, has not been described before. In a previous work, our group found that chronic IGE patients taking a heavier load of ASM had lower beta 1 ImCoh. In that cohort, the decrease in FC was present in a bilateral and widespread pattern.^25^ In the present study, newly diagnosed, drug‐naïve patients exhibited a decrease in beta 1 activity mainly in the right hemisphere with the strongest effect size in frontotemporal regions (*d* > 0.08). In the left hemisphere, the pattern did not survive correction (*P*_uncorr_ <0.05, *d* < 0.08) but had an intermediate to large effect size in the same areas. The differences in the pattern in comparison with the mentioned study,^25^ could be related to the different stages of the disease in which each cohort was evaluated. This suggests that the beta 1 decrease might be attributed to factors beyond just the ASM treatment. Given that the EEGs were recorded on average 45 days after the ictal event, it is not likely that this decrease is caused by postictal mechanisms, as these are estimated to typically last up to 24 h after the seizure.^55^ Considering the large effect size, the observed FC decreases could be the measurable outcome of a compensatory mechanism within the brain, aimed at mitigating the risk of subsequent seizures, which in the group of single‐seizure patients was successful at least during the follow‐up period. However, this initial observation needs to be evaluated in further studies with longer follow‐ups and more subjects.

Previous studies have investigated epilepsy biomarkers through resting state EEGs, comparing patients with epilepsy to those suspected of having a single seizure.^34^, ^36^, ^56^ One such study reported increased theta FC in patients who developed epilepsy and also identified it as a sensitive (62%) and specific (76%) predictor of epilepsy.^34^ This study used synchronization likelihood (SL) as the metric of FC. van Diessen et al.^36^ also applied SL in a broadband approach within a prediction model to identify children with partial epilepsy from a group of patients suspected of having a first seizure. This model had a sensitivity of 96% and a specificity of 95%. Another study, which employed a machine learning classifier for epilepsy diagnosis in patients after a first ictal episode, found that among a cohort of 291 epilepsy patients and 213 patients with other pathologies, FC had the highest predictive power among the evaluated features (74% sensitivity, 57% specificity).^56^ Interestingly, delta‐related features played a considerable role in the models.^56^ In contrast to these studies, our results indicate that delta FC is lower in the epilepsy group compared to the single‐seizure patients.

An important consideration is the different patient profiles in the mentioned studies. In particular, in the study of Douw et al.,^34^ their epilepsy cohort included patients with IEDs, radiological abnormalities, and CNS active medication, while their single‐seizure group comprised patients with a first seizure that also had conditions like neuropathy, syncope, migraine, and psychogenic causes. All of which could have an effect on FC. Methodologically, they employed synchronization likelihood (SL) in sensor space, contrasting with our use of ImCoh in source space, which mitigates potentially spurious instantaneous interactions due to field spread.^45^ These multiple differences hinder a direct comparison, but it is possible that theta FC changes may be due to radiological abnormalities and/or related to IEDs. Conversely, our non‐lesional, IED‐free, and unmedicated cohort suggests that the observed changes in ImCoh and power are not attributable to these factors.

The finding of lower delta FC in the epilepsy group, compared to single‐seizure patients and controls, showed a unilateral pattern only apparent in the left hemisphere. This might be explained by the fact that 83.3% (*n* = 10) of the patients with focal epilepsy had presumed left‐sided epileptogenesis. Although, we did not find differences in the subanalysis between the patients with left epileptogenesis (*n* = 10) against controls (data not shown). We believe the small sample size hindered these comparisons, as previous studies have shown that patients with focal epilepsy exhibit changes in the epileptogenic hemisphere.^50^, ^57^, ^58^ Considering that our cohort was non‐lesional, the lack of differences when comparing the ipsilateral and contralateral hemispheres of the focal epilepsy patients (Figure S1A; Table S2) aligns with previous research,^31^ which shows widespread changes in power and ImCoh.

The delta and theta band alterations in the epilepsy group suggest a possible widespread network reorganization linked to early disease stages. Delta oscillations, thought to enable long‐range communication,^59^ may reflect network changes during disease progression, supported by the observed increase in theta and delta power, which reflects a hyperexcitable brain.^34^ Further, the decreased beta 1 FC in the single‐seizure group suggests predominantly local changes.^59^ Our results suggest that initial changes to a first seizure involve increased slow‐wave power and reduced long‐range communication, particularly in the delta band, indicating a shift toward a hyperexcitable network.

Unlike healthy subjects, the epilepsy group showed increased theta FC in occipital, parietal, and temporal regions, along with widespread power increases in theta and delta frequencies. Previous studies on established epilepsies typically report widespread increases in power from delta to gamma bands^24^, ^25^, ^26^, ^27^, ^60^ and in theta, alpha, and beta1 in FC for IGE patients.^25^, ^31^, ^61^, ^62^ Regarding focal epilepsies, there are mixed results of FC and power in the theta, beta, and alpha frequencies.^28^, ^29^, ^31^, ^50^, ^57^, ^58^, ^61^, ^62^ In contrast, we found changes only in theta and delta frequencies. This could be explained as our epilepsy group included patients with both focal (temporal and frontal) and generalized epilepsy. In the subanalysis between these subgroups, patients with focal epilepsy (*n* = 5) showed higher beta 1 FC in the right hemisphere (Figure S1B; Table S1), while the IGE patients (*n* = 12) had higher theta FC in the right temporal lobe (Figure S1B; Table S1). We believe that changes were found only in theta and delta frequencies because alterations in these frequencies are characteristic of both types of epilepsy.^24^, ^25^, ^26^, ^58^, ^63^, ^64^ In contrast, changes in other frequencies may be more cohort‐dependent.

Delta and theta rhythms during wakefulness are classically associated with neurological disorders and damage.^65^, ^66^ This aligns with our findings, as the changes we observed are likely caused by alterations already present at early stages of the disease. This corresponds with findings from a similar cohort of unmedicated, newly diagnosed IGE patients.^24^, ^67^

Traditionally, studies of brain network changes in epilepsy have focused on focal and generalized types separately due to differing pathophysiologies. However, recent research suggests that a genetically determined seizure liability, known as epileptic diathesis, influences all epilepsy types.^63^ This theory is supported by studies involving both patients and their asymptomatic siblings.^25^, ^68^ We propose that altered cortical excitability can be observed after the first seizure, regardless of the epilepsy type.

Speculating on the neurobiological mechanisms behind our findings is beyond this study's scope, but thalamic dysregulation, previously suggested for IGE,^61^ may play a role. Thalamic nuclei, crucial for modulating long‐range synchronizations in both focal and generalized epilepsies,^69^, ^70^ and thalamocortical connectivity, implicated in seizure generation and treatment^71^, ^72^, ^73^; might influence network changes via slow oscillations in early‐stage epilepsy.

Our results support the hypothesis that FC and power networks differ significantly between patients likely to develop epilepsy after a first seizure and those who do not. Moreover, patients who remained seizure‐free exhibited distinct FC patterns from healthy controls, which may be associated with a reduced likelihood of experiencing further seizures.

## 5 LIMITATIONS

This retrospective study has limitations, including the inability to track diagnoses beyond 6 months, which may result in missing long‐term seizure occurrences. Additionally, relying on hospital clinical reports for medication data and medical history may introduce biases and lead to incomplete information. Another limitation, is that the clinical EEG was restricted to 19 channels, which may impact the spatial precision of source localization.^74^ However, there is growing evidence that source‐reconstruction of ictal and interictal epileptic activity from low‐density EEG is both feasible and clinically informative.^38^, ^63^, ^64^, ^75^, ^76^ Additionally, recent studies have shown that similar FC results are found when comparing datasets from HD‐EEG and routine‐EEG in the same group of participants.^37^, ^77^

### 5.1 Future directions

As this is an exploratory, retrospective study, future prospective studies should replicate these findings with a larger cohort, individual MRI images for source reconstruction. In addition, expanding the comparison to non‐epileptic patient groups, such as migraine or syncope, in future studies would provide a more comprehensive clinical framework and strengthen the diagnostic relevance of the findings.

## CONFLICT OF INTEREST STATEMENT

NKF received honoraria from Arvelle, Jazz Pharma, Bial, Eisai, and Precisis, as well as research support from Jazz Pharma, all unrelated to this work. EHUR received honoraria from Jazz Pharma, Eisai, and Springer, all unrelated to this work. The remaining authors have no conflicts of interest.

## ETHICS STATEMENT

We confirm that we have read the Journal's position on issues involved in ethical publication and affirm that this report is consistent with those guidelines.

## Supporting information


**Figure S1.** Electroencephalography analysis of power and functional connectivity of patients that developed focal or generalized epilepsy.

**Table S1.** Group level statistics of vertex power and connectivity.

**Table S2.** Power and ImCoh global comparisons between ipsilateral and contralateral hemispheres of patients with focal epilepsy.

## Data Availability

The data that support the findings of this study are available on request from the corresponding author. The data are not publicly available due to privacy or ethical restrictions.
