# Supplementary material for: Quantitative EEG signatures in patients with and without epilepsy development after a first seizure
Source: Epilepsia Open. 2025 Mar 4;10(2):427–40. doi: 10.1002/epi4.13128 (PMC12014921; doi:10.1002/epi4.13128)

Figure S1

A) Focal epilepsy sub-analysis: Ipsilateral hemisphere vs contra-lateral hemisphere

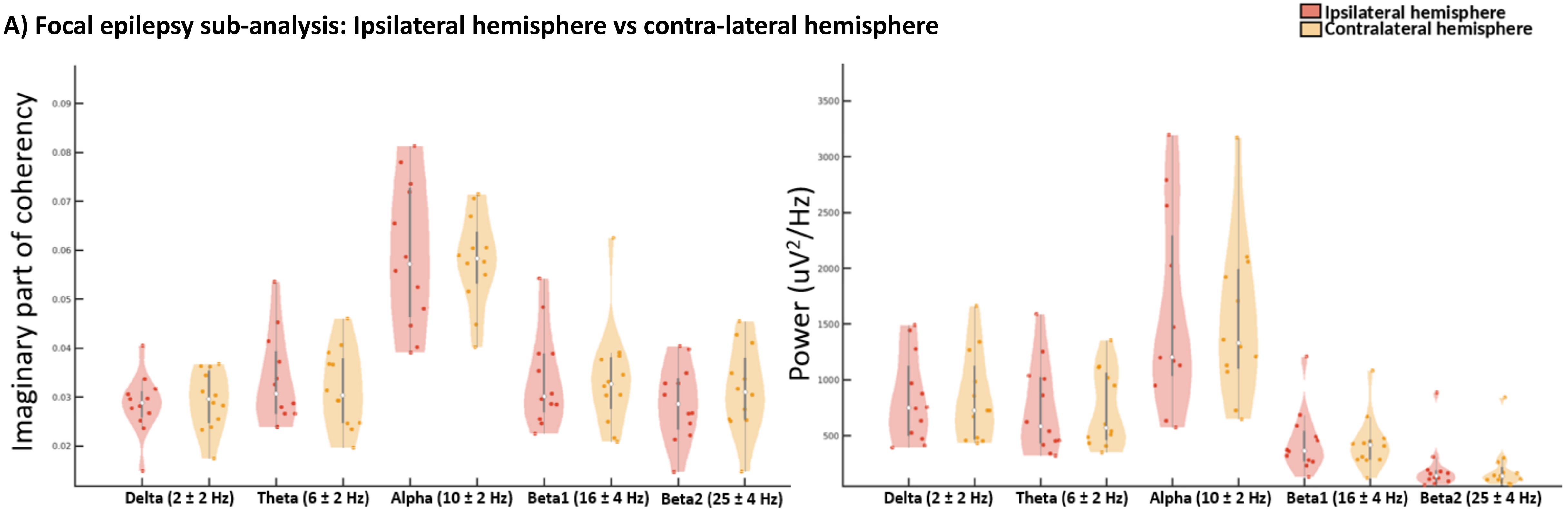

B) Comparison between focal epilepsy patients vs IGE patients

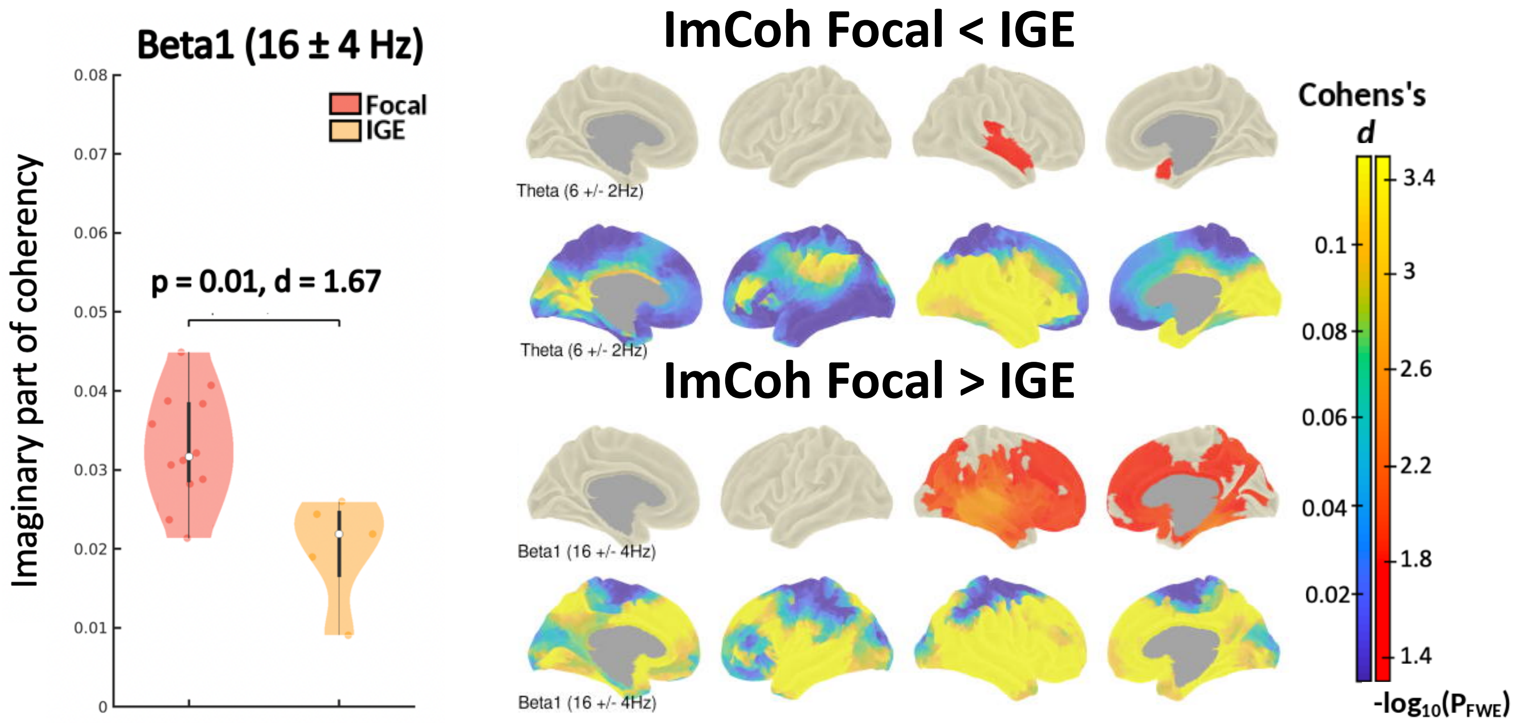

Supplement: Supplementary file 1 — Figure S1. Electroencephalography analysis of power and functional connectivity of patients that developed focal or generalized epilepsy. [file EPI4-10-427-s001.pdf]
